# Structure of actomyosin rigour complex at 5.2 Å resolution and insights into the ATPase cycle mechanism

**DOI:** 10.1038/ncomms13969

**Published:** 2017-01-09

**Authors:** Takashi Fujii, Keiichi Namba

**Affiliations:** 1 Graduate School of Frontier Biosciences, Osaka University, and Riken Quantitative Biology Center, 1-3 Yamadaoka, Suita, Osaka 565-0871, Japan

## Abstract

Muscle contraction is driven by cyclic association and dissociation of myosin head of the thick filament with thin actin filament coupled with ATP binding and hydrolysis by myosin. However, because of the absence of actomyosin rigour structure at high resolution, it still remains unclear how the strong binding of myosin to actin filament triggers the release of hydrolysis products and how ATP binding causes their dissociation. Here we report the structure of mammalian skeletal muscle actomyosin rigour complex at 5.2 Å resolution by electron cryomicroscopy. Comparison with the structures of myosin in various states shows a distinctly large conformational change, providing insights into the ATPase-coupled reaction cycle of actomyosin. Based on our observations, we hypothesize that asymmetric binding along the actin filament could function as a Brownian ratchet by favouring directionally biased thermal motions of myosin and actin.

## Abstract (web summary)

The cyclic association and dissociation of myosin with actin filament is regulated by ATP binding and hydrolysis cycles. Here the authors report the structure of mammalian skeletal muscle actomyosin rigour complex that provides insights into the ATPase-coupled reaction cycle of actomyosin.

Molecular motors are nanomachines that convert chemical energy to mechanical work for unidirectional movement. Actomyosin motors have been extensively studied over more than half a century, as contractile elements of muscle in early stages and also as elements driving intracellular transport in recent years^1^,^2^,^3^,^4^,^5^. Muscle contraction occurs through mutual sliding of thick myosin filaments and thin actin filaments that shorten sarcomeres, the contractile units regularly repeating along muscle cells^6^. The sliding force is generated via cyclic interactions of myosin heads projecting from the thick filament with actin molecules on the thin filaments. Myosin head is an ATPase, and its ATP binding and hydrolysis regulates the cyclic association and dissociation of myosin with actin filament^7^. Upon binding of MgATP, myosin hydrolyses ATP relatively quickly but the hydrolysis products ADP and Pi stay in the nucleotide-binding pocket, and therefore its ATPase cycle does not proceed until myosin head binds to actin filament. A conformational change of myosin head upon binding to actin filament must be responsible for this actin-activated ATPase, but structural information on the actomyosin rigour complex is still limited to reveal the mechanism. Structural studies by X-ray crystallography on the head domains of various myosins, such as myosin II, V and VI, in different nucleotide states have suggested that myosin undergoes large conformational changes during ATPase cycle in its lever arm domain to be in largely different angles within the plane of actin filament axis and that such changes represent a power stroke that drives the unidirectional movement of myosin against actin filament^1^,^2^. However, as those myosin head structures obtained in atomic details are all in the absence of actin filament^8^,^9^,^10^,^11^,^12^,^13^,^14^,^15^,^16^,^17^, key piece of information is still missing.

The structure of the actomyosin rigour complex has been analysed by electron cryomicroscopy (cryoEM) and image analysis^18^,^19^. However, the resolution and quality of the density maps were limited to reveal the conformational changes in sufficient detail, and it was still not so clear how ADP and Pi are released upon strong binding of myosin to actin filament and how ATP binding to myosin causes its dissociation from actin filament. Here we report a cryoEM structure of the actomyosin rigour complex of rabbit skeletal muscle at 5.2 Å resolution. Comparison of this structure with those of myosin in various states now reveals a distinctly large conformational change that widely opens up the nucleotide-binding pocket to release ADP and Pi upon binding of myosin to actin filament and how actomyosin dissociates upon ATP binding for the next reaction cycle for sliding force generation.

## Results

### CryoEM data collection and helical image analysis

For sample preparation of the actomyosin rigour complex, we used apyrase to completely remove residual ATP in solution to stabilize the rigour complex. Frozen-hydrated specimen grids were prepared by Vitrobot (FEI) and observed at temperatures of 50–60 K using a JEM-3200FSC electron cryomicroscope (JEOL) with a liquid-helium cooled specimen stage, an Ω-type energy filter and a field-emission electron gun operated at 200 kV. Zero energy-loss images were recorded on a TemCam-F415MP 4 k × 4 k CCD camera (TVIPS) at an approximate magnification of × 111,000 (1.35 Å per pixel), a defocus range of 1.0–2.0 μm and an electron dose of ∼20 electrons per Å^2^. In total, 779 CCD images were collected manually in 3 days and used for image analysis.

Helical image analysis was carried out using the IHRSR (iterative helical real-space reconstruction) method^20^ with EMAN (Electron Micrograph ANalysis)^21^ and SPIDER^22^ as previously described^23^,^24^. Images of actomyosin filament in 779 CCD frames were boxed into 31,535 segments of 512 × 512 pixels with a step shift of 100 pixels along the filament axis using the boxer program of EMAN^21^. The number of actin–myosin molecules used for three-dimensional (3D) image reconstruction corresponds to ∼120,000. The statistics of data collection and image analysis is given in Supplementary Table 1. The helical image analysis produced a well-defined 3D density map of the actomyosin rigour complex at 5.2 Å resolution (at a Fourier shell correlation of 0.143, Supplementary Fig. 1). Most of the secondary structures of actin and myosin observed in their crystal structures were clearly identified in the 3D map, indicating the high quality of the map that assures the reliability of the fitted and refined atomic model.

### CryoEM map and fitted model of the rigour complex

**Figure 1:**
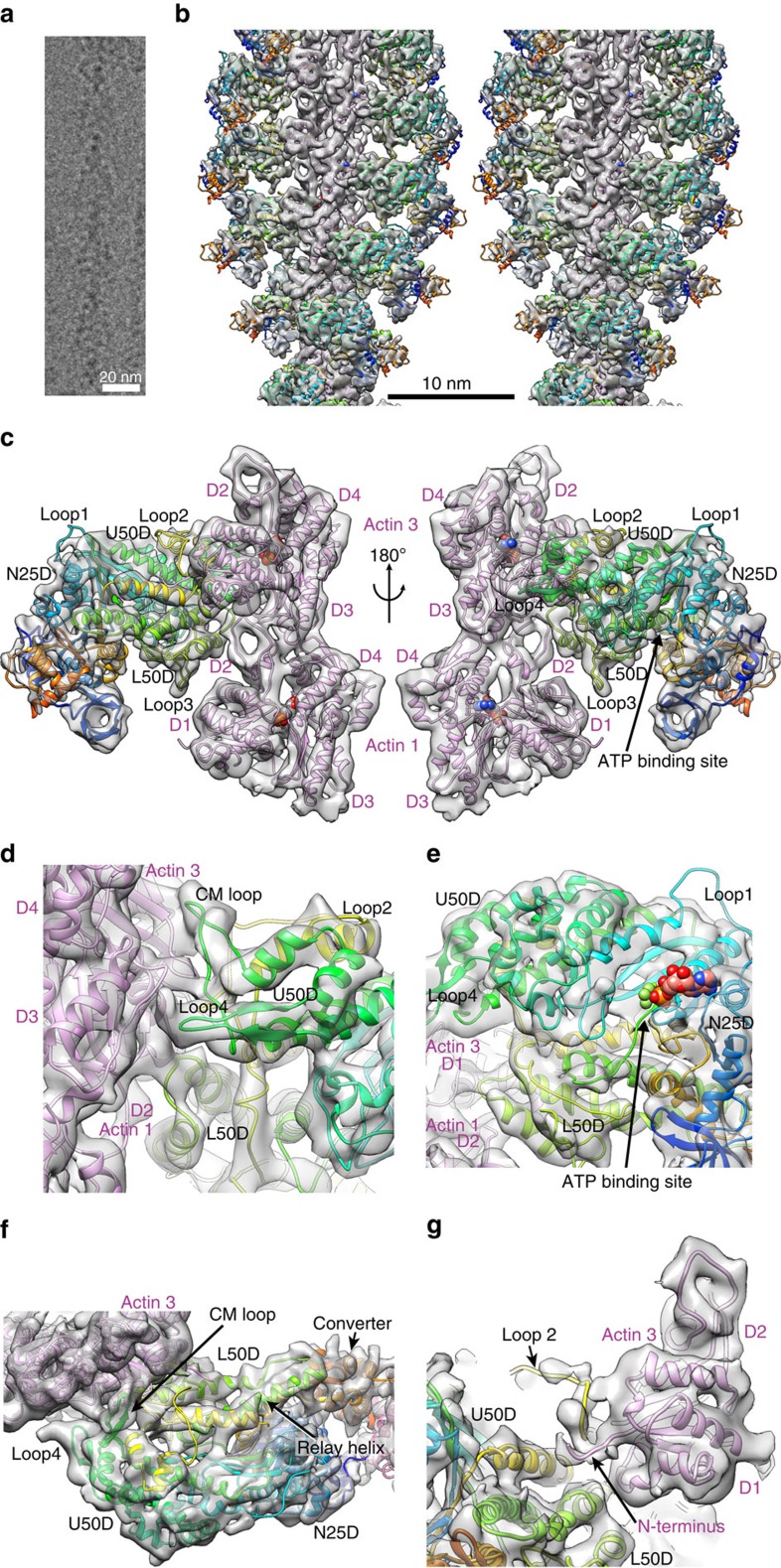
CryoEM density map and fitted model of actomyosin rigour complex. (**a**) A cryoEM image showing the typical arrowhead feature of the complex. (**b**) The 3D density map and fitted model of actin filament fully decorated with myosin heads. Approximately 10 subunits of actin and myosin head are shown. Ribbon models of actin are coloured purple and myosin in rainbow according to the sequence in **b**–**g**. (**c**) Two magnified views in front and back showing intimate interactions of a myosin head and two actin subunits along one strand of actin filament. Actin subunits are numbered 1 and 3 along the 1-start helix of actin filament. (**d**) A view similar to the right panel of **c** but magnified and further rotated to show the contact of CM loop and loop 4 with actin. (**e**) A view similar to the right panel of **c** but magnified and viewed from a lower viewpoint to show the widely open ATP-binding pocket. MgADP-BeF_3_ from a post-rigour myosin structure (PDB: 2VAS)^17^ is shown in CPK as a guide, with BeF_3_ in green. Near the arrowhead are P-loop and Switch-2. (**f**) An end-on view from pointed end of actin filament showing the interactions of myosin with actin: CM loop and loop 4 on the left; L50D and loop 2 on the right. (**g**) The same view as the left panel of **c** but further magnified with the map contoured at a slightly lower level and shown in a thinner slab to clearly show the density of myosin loop 2 and the N-terminal chain of actin.

The structure of the skeletal actomyosin rigour complex is shown in Fig. 1 (Supplementary Movies 1 and 2) together with a typical cryoEM image of F-actin fully decorated with myosin heads (Fig. 1a). The helical symmetry and axial repeat distance were refined and converged to a subunit rotation of −166.67° and an axial repeat of 27.6 Å. These are identical to those of F-actin^23^, indicating that subunit packing interactions of actin molecules are not affected by myosin head binding.

The density map presented in stereo (Fig. 1b) shows a short segment containing ∼10 subunits of actin (purple) and myosin head (rainbow) with their atomic models. Most of the secondary structures, such as α-helices, β-sheets and loops, are clearly resolved for both actin and myosin, allowing reliable model fitting and refinement (Fig. 1b–e and Supplementary Movie 2). We used a homology model of rabbit skeletal muscle myosin based on the crystal structure of squid muscle myosin S1 fragment in the rigour-like state (Protein Data Bank (PDB): 3I5G)^16^ and a cryoEM structure of F-actin from rabbit skeletal muscle (PDB: 3MFP)^23^ for docking and refinement by flexible fitting^25^,^26^. We carried out this model fitting refinement carefully to avoid overfitting, by imposing a relatively strong restraint to keep the conformations of individual domains with independent hydrophobic cores unchanged as much as possible and trying not to fit individual secondary elements separately, just like we did for the actin filament structure^23^. As a reliability measure of our model, the root mean square (r.m.s.) deviations of Cα atoms for individual domains of myosin head between our refined rigour model and crystal structures of different states are listed in Supplementary Table 2. The r.m.s. deviations of our model from the crystal rigour-like structure (PDB: 3I5G)^16^ are all within a range from 1.0 to 1.6 Å and comparable to those between the crystal structures, assuring that our model was refined without overfitting.

The F-actin model fitted well into the density map without any modification, and further refinement did not show any significant changes except for its N terminus. The homology model of myosin head in the rigour-like conformation did not fit well into the map (Supplementary Figs 2 and 3), and therefore significant rearrangements of domains and secondary structures by flexible fitting were necessary to refine the model against the map. The quality and reliability of this atomic model can be assessed by the quality of model fit to the map (Supplementary Movie 2) and also by comparing it with the fit of the crystal rigour-like model to the map (Supplementary Figs 2 and 3) as well as the one by the previous study on the cryoEM actomyosin rigour structure at 8 Å resolution^19^ (Supplementary Fig. 4).

Figure 1c shows two side views, in front and back, of a myosin head attached to two actin molecules along one of the two long strands of F-actin. Side view figures in this paper are all presented with the pointed end of F-actin up. Magnified views of actomyosin contact and the nucleotide-binding site are presented in Fig. 1d,e, respectively. Figure 1f is a view from the pointed end nearly along the F-actin axis where one myosin head is displayed on the right-side strand of F-actin. Myosin head can be roughly divided into the following five subdomains: the N-terminal 25 kDa domain (N25D: 1–205); the upper 50 kDa domain (U50D: 206–466; 603–627); the lower 50 kDa domain (L50D: 467–602; 628–680); the converter domain (CD: 681–770); and the lever arm domain (LAD: 771–845) composed of the long C-terminal α-helix wrapped around by the essential and regulatory light chains. The secondary structures of the core of myosin motor domain, formed by N25D, U50D, L50D and CD, are well resolved including the SH3 domain (D33–M80), loop 3 (K567–F579), loop 4 (K365–G379) and cardiomyopathy (CM) loop (C403–Q417) on the surface. However, the densities for the N-terminal chain connecting to SH3 (E26–F32), loop 1 (K206–G216) and an N-terminal portion of loop 2 (N625–G635) are not visible because of disorder (Fig. 1c,g, Supplementary Movie 2). The C-terminal chain is well resolved up to the SH1–SH2 helices, but part of the converter domain is out of the density, and the C-terminal long helix is clearly visible only up to L784 whereas the S1 fragment contains 845 residues. The densities of further C-terminal chain as well as the essential and regulatory light chains that form the lever arm are also visible but are too weak to build a reliable model. The domains and loops are coloured and labelled in Supplementary Fig. 5 as a guide.

### Intermolecular interactions in the actomyosin rigour complex

**Figure 2:**
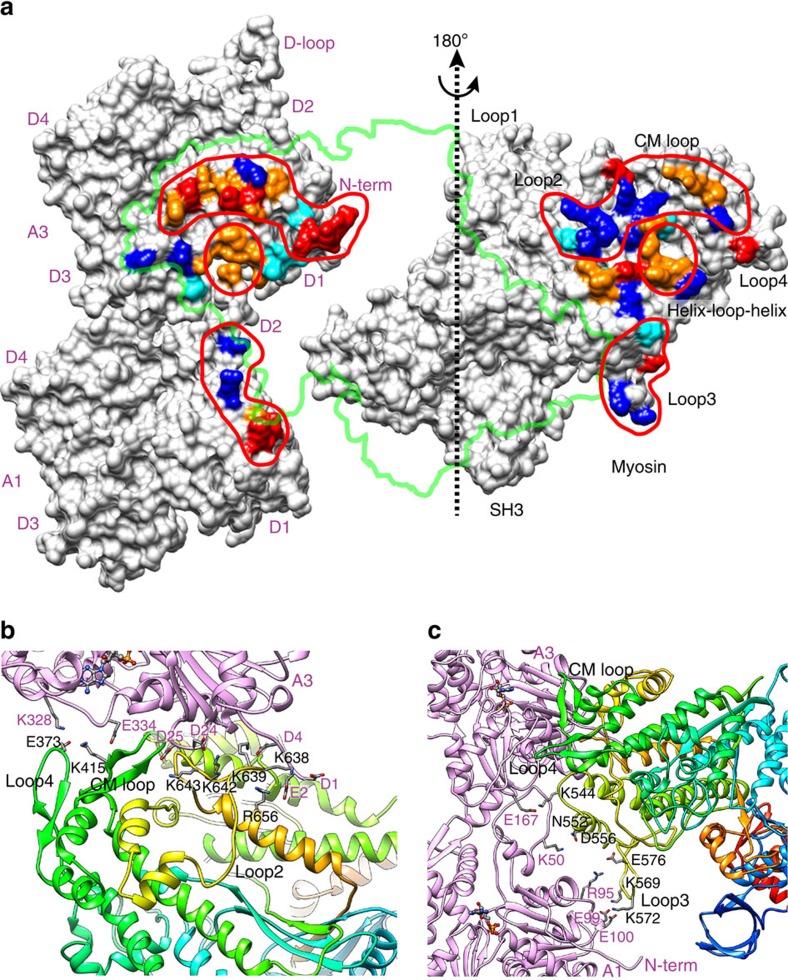
Characteristics of actin and myosin interactions. (**a**) Myosin head is rotated 180° to show the nature of each interacting surface area. The thick green line indicates the shape and position of myosin head in the rigour complex. Actin subunits are numbered A1 and A3 as in Fig. 1c. Colours indicate negative charge in red, positive charge in blue, polar in light blue and hydrophobic in brown. (**b**) End-on view from the pointed end as Fig. 1f, showing side chains involved in electrostatic interactions. Interactions between clusters of negative charges of actin N-terminal region and positive charges of myosin loop 2 play essential roles in weak binding. (**c**) Side view showing another region of electrostatic interactions: myosin K544 and D556 of L50D helix–loop–helix could form salt bridges with A3:E167 and A1:K50, respectively; K569, K572 and E576 of myosin loop 3 could form salt bridges with E99, E100 and R95 of A1, respectively. Note that the side-chain conformations shown here are not experimentally supported because of the limited resolution of the 3D map.

Each myosin head interacts with two actin molecules along one of the two long strands of F-actin (Figs 1c and 2) as described in the previous cryoEM studies^18^,^19^. We can now see their interactions in much more detail with accuracy at a level of amino-acid residues involved in the interactions because the main chain positions are much more accurate and reliable than those of the previous studies, although caution should be taken that the side-chain conformations depicted in Fig. 2 are not experimentally validated. Actin subunits in Fig. 2a are numbered A3 and A1 from the pointed end. Three major myosin-binding sites are identified on the F-actin surface. One is a hydrophobic patch (brown in Fig. 2a) formed by the bottom part of domain D1 of A3 (Y143, I345, L346, L349, F352) and the DNase-I-binding loop (D-loop) of A1 (M44–M47). The tip of the helix–loop–helix structure (I532–H558) of myosin L50D binds to this site with conserved residues M541, F542 and P543. There are also electrostatic interactions below these hydrophobic ones (M:K544–A3:E167, M:N552/D556–A1:K50), further stabilizing the actomyosin complex. The second one is located in domain D1 of A3 just above this hydrophobic patch. A mixture of charged and apolar residues of actin form hydrophobic interactions with those of CM loop of myosin U50D (M:Y412–A3:Y337/E334, M:V408–A3:A16/P27, M:K415–A3:P333). The third one is located in domains D1 and D3 of A3 above left of the hydrophobic patch. Glu373 at the tip of loop 4 of myosin U50D forms electrostatic interactions with Lys328 of A3.

Additional, yet important, interactions with actin are found in myosin loop 2 connecting U50D and L50D (N625–F648). The map shows a density for its C-terminal part (S637–F648) with multiple lysine residues interacting with actin (Fig. 1g). Two lysine residues of myosin are closely located to negative charges of actin N terminus (M:K638–A3:D1/E2, M:K639–A3:E4) and another pair of lysine residues are interacting with two actin residues (M:K642–A3:D24, M:K643–A3:D25), forming intimate electrostatic interactions (Fig. 2b). Another salt bridge (M:E656–A3:E2) also contributes to this charge interaction. The density of the actin N-terminal chain is relatively weak but shows its position slightly down from that of F-actin^23^ by a few Å (Fig. 1g). This is the only part of actin that showed a clear conformational change upon myosin binding.

Myosin loop 3 (K567–F579), which was thought to form an extensive interaction with residues 95–100 of actin A1 (ref. 27), does not appear to interact with actin so extensively (Fig. 1e) although contributing to electrostatic interactions to some extent (M:K569–A1:E99, M:K572–A1:E100; M:E576–A1:R95) (Fig. 2c). Lorenz and Holmes^28^ carried out MD simulations of the actomyosin model based on a cryoEM density map at 13 Å resolution^18^ and described actomyosin interactions in detail, but none of the residue pairs between actin and myosin they described were found in our structure except for those between myosin loop 3 and actin residues 95–100 listed above. However, as the amino-acid sequence of loop 3 varies with the type of myosin, its interaction with actin is also likely to be variable, affecting the kinetic parameters that determine the characteristic differences of myosin motor functions^29^.

### Conformational differences of myosin in different states

**Figure 3:**
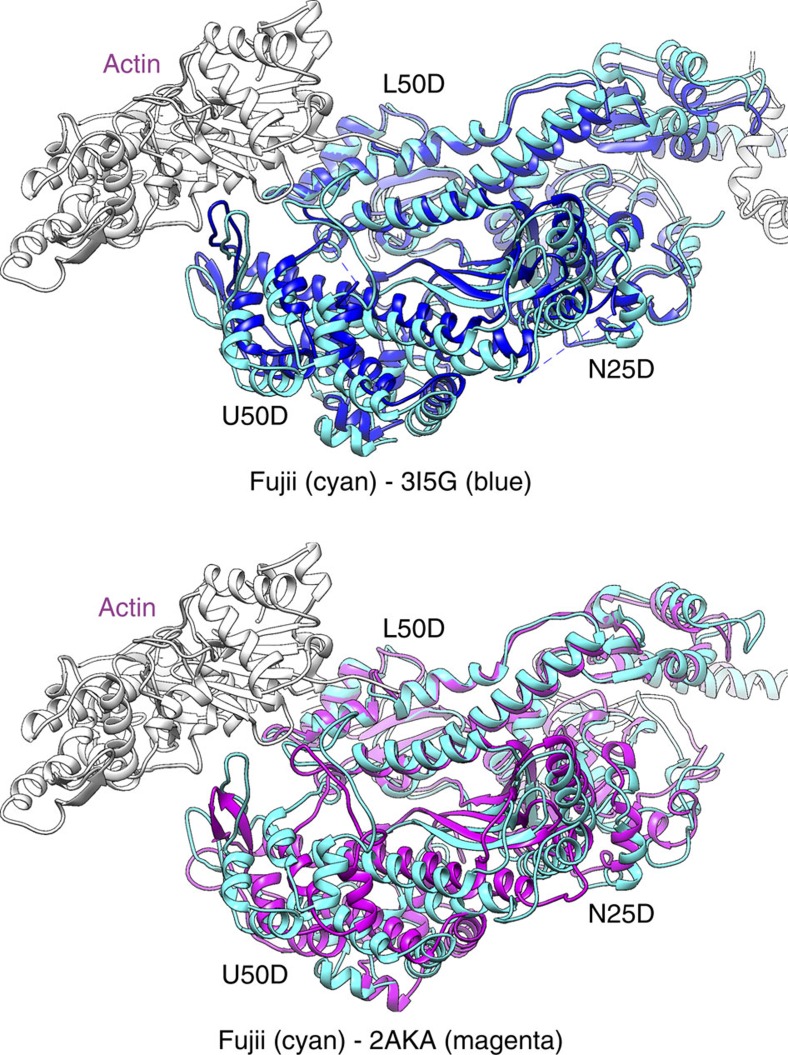
Comparison of the rigour and rigour-like structures. Axial views from the pointed end showing the actomyosin rigour model with two rigour-like crystal structures (PDB: 3I5G^16^ and 2AKA^13^,^14^). L50D is used for superposition. Molecules are coloured as follows: actin in grey; rigour myosin in cyan; rigour-like myosins in blue (3I5G) and magenta (2AKA).

Crystal structures of many different myosins in various nucleotide states have been classified into three distinct conformations: rigour like, post-rigour and pre-power stroke^16^. We compared the structure of myosin in the rigour complex with the crystal structures of myosin in the nucleotide-free rigour-like state (for example, PDB: 3I5G^16^ and 2AKA^13^,^14^: myosin II; 1OE9^12^: myosin V; 2BKI^15^: myosin VI), those in the pre-power stroke state (for example, PDB: 1BR1^9^: myosin II; 1QVI^30^: myosin II) and those in the post-rigour state (for example, PDB: 2MYS^8^: myosin II) to see their conformational differences. We first compared our rigour structure with the crystal rigour-like structures by superposing residues 470–560 of L50D that form the relay helix and the helix–loop–helix bound to two actin subunits (Fig. 3). Although overall conformation is similar between them, N25D and U50D both show significant differences in position and orientation, producing either steric clashes or gaps between U50D and actin in the rigour-like structures. Although the cleft between U50D and L50D is closed in the rigour-like structure compared with those in the post-rigour and pre-power stroke states, the cleft seems to be slightly more open in the actomyosin rigour structure. The position and orientation of myosin U50D relative to L50D in the rigour state compared with those in the post-rigour and pre-power stroke states is more typically characterized as a clockwise swing by ∼20° in the rigour state, as shown in Supplementary Movie 3.

**Figure 4:**
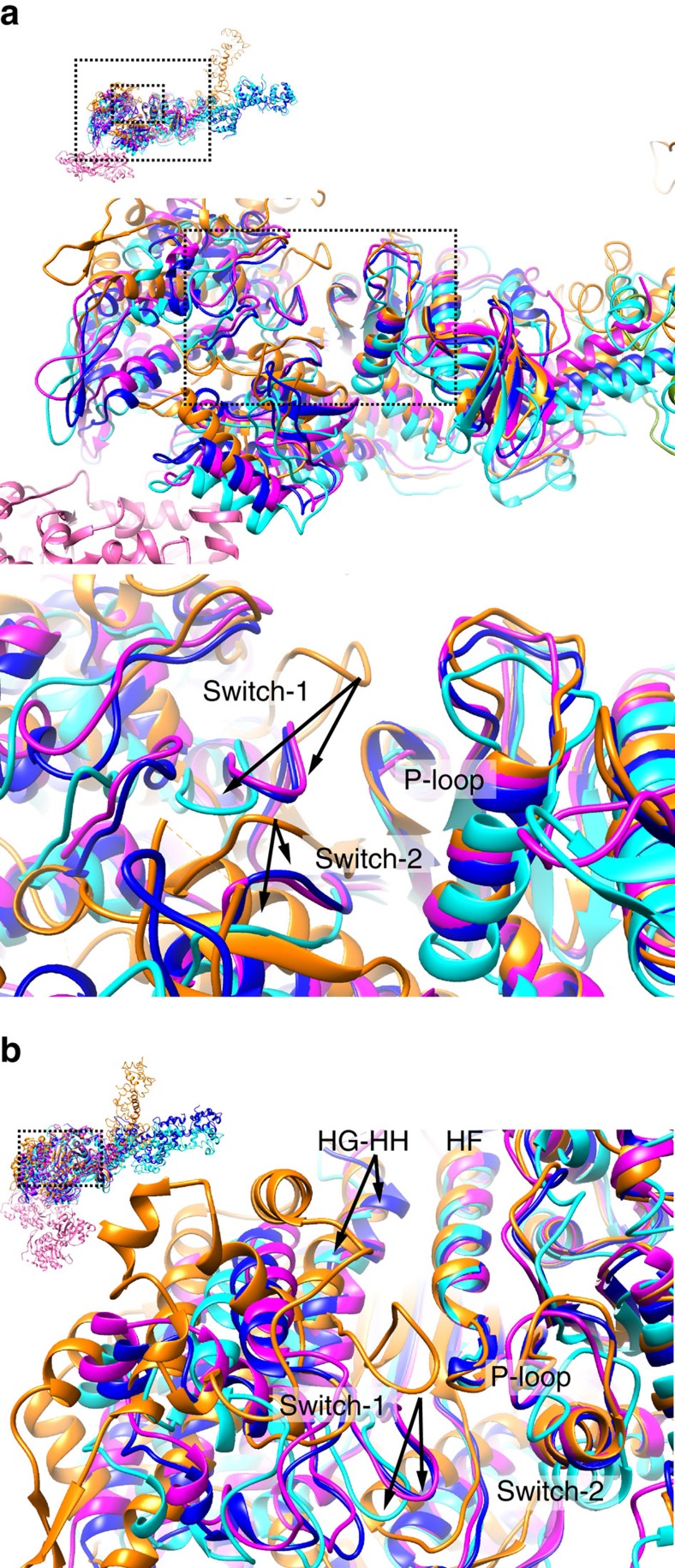
Comparison of myosin structures in the actomyosin rigour state and in the rigour-like and post-rigour states in crystals. (**a**) Four different models of myosin II superposed with P-loop-containing strand-helix motif of N25D, and their nucleotide-binding sites viewed from the barbed end of actin filament coloured pink: cryoEM rigour in cyan; rigour-like (PDB: 2AKA)^13^,^14^ in magenta; rigour-like (3I5G)^16^ in blue; post-rigour (2MYS)^8^ in orange. The small figure with dotted boxes is a guide for an enlarged overview in the middle and a further magnified view at the bottom. (**b**) Same as **a** but viewed more obliquely to show how widely open the nucleotide-binding pocket is in the rigour state. Black arrows indicate the positional changes of Switch-1 and Switch-2 in **a**,**b**.

This clockwise U50D motion together with a concomitant counterclockwise motion of N25D widely opens up the nucleotide-binding site, and this can be seen more clearly by superposing N25D. The atomic models of myosin head in different states were superposed with residues R170–A200 of rabbit myosin II, which form the fourth strand of the seven-stranded β-sheet, P-loop and helix HF, to make P-loop in the N-terminal domain as the reference to see the movements of other domains. The entire N-terminal domains of myosins were all well superposed by this. ATP and its hydrolysis products ADP and Pi are coordinated by residues of P-loop, Switch-1 and Switch-2 in the post-rigour and pre-power stroke states, respectively, but in the rigour-like state Switch-1 and Switch-2 both move away from P-loop (Fig. 4a). In the actomyosin rigour state, they move further away from P-loop (Fig. 4a) by a distinctly larger movement of U50D than those seen in the rigour-like state, making the nucleotide-binding pocket between helices HF and HG-HH more widely open (Fig. 4b and Supplementary Fig. 3). The structure of myosin I bound to actin filament with tropomyosin in the rigour complex solved by a recent cryoEM study (PDB: 4A7F)^19^ somehow showed the positions of Switch-1 and Switch-2 quite far from those in our rigour structure and even from those of rigour-like myosin structures (Supplementary Fig. 6). Hence, the conformation of myosin in the actomyosin rigour state we report here is distinct from any of the rigour and rigour-like structures previously reported.

**Figure 5:**
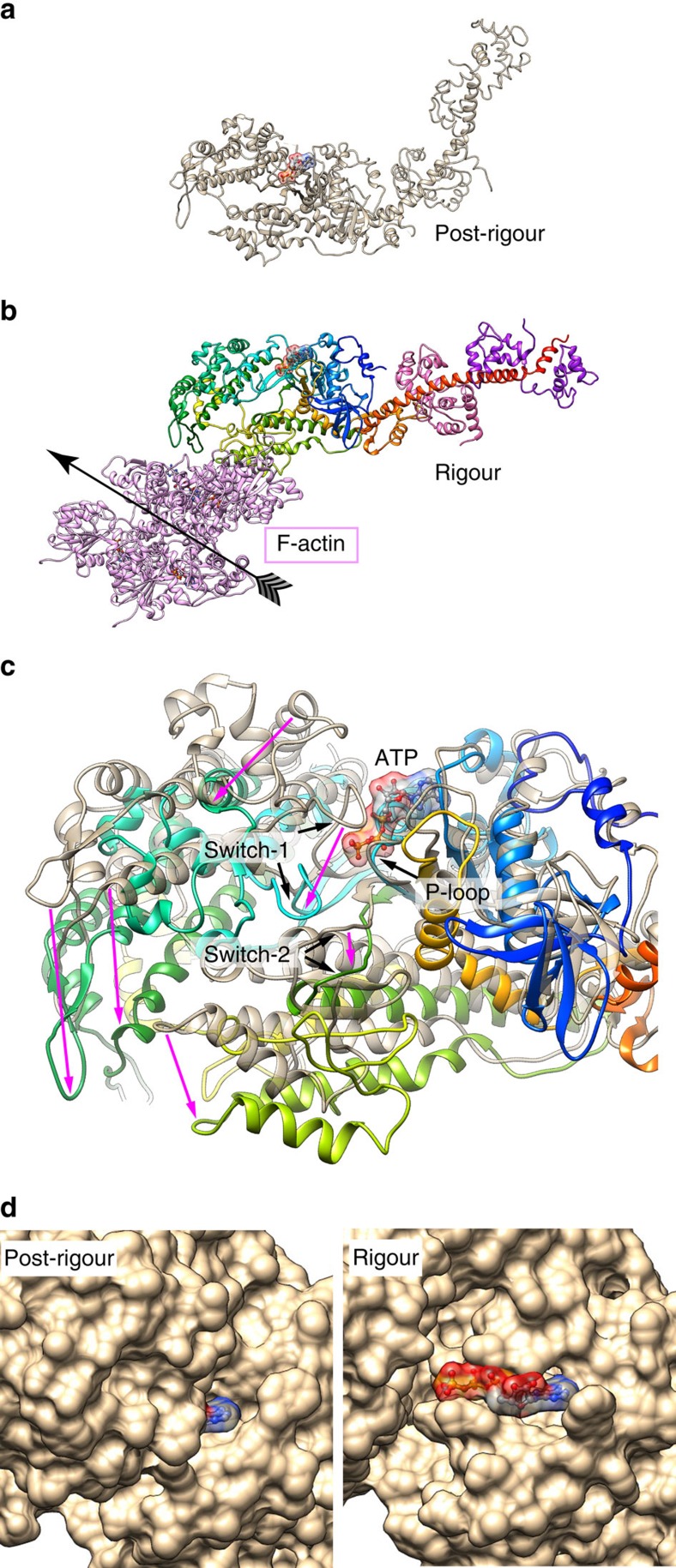
Comparison of myosin structures in the actomyosin rigour state and a post-rigour state. (**a**) The post-rigour crystal structure of chicken muscle myosin (2MYS)^8^. (**b**) The actomyosin rigour complex nearly in end-on view from the barbed end of actin filament. ATP is included in both models to indicate its binding position. (**c**) The models in **a**,**b** are superposed as in Fig. 4a to show the motions of U50D and L50D relative to N25D that causes wide opening of the nucleotide-binding pocket. (**d**) The nucleotide-binding sites of the two models in solid surface representation showing how widely the nucleotide-binding pocket is open when myosin head is bound strongly to actin filament.

We further compared our rigour structure with one of the post-rigour structures obtained from the crystal of nucleotide-free myosin II from chicken skeletal muscle^8^, with MgATP placed as a guide in the nucleotide-binding site according to the structure of MgATP-bound *Dictyostelium discoideum* myosin^10^ (Fig. 5 and Supplementary Fig. 7). As the nucleotide-binding pocket in the pre-power stroke state shows nearly the same closed conformation to that in post-rigour state, the comparison between the rigour and post-rigour states also reveals the structural change around the nucleotide-biding pocket upon binding of myosin in the ADP–Pi state to actin filament. This comparison revealed how largely Switch-1 and Switch-2 move away from the phosphate moiety in the rigour state to widely open the nucleotide pocket to expose the entire nucleotide including Pi (Fig. 5c,d).

We compared our structure with a recent cryoEM structure of a human cytoplasmic actomyosin rigour complex at 3.9 Å resolution^31^. Although the interactions between two actin subunits and myosin head are very similar to each other (Supplementary Fig. 8a), the nucleotide-binding pocket of the human cytoplasmic myosin is similar to those of crystal rigour-like structures and appears more closed than that of the skeletal actomyosin rigour structure reported here (Supplementary Fig. 8b–d), whereas overall individual domain conformations are nearly identical (Supplementary Table 3). Thus, our 5.2 Å resolution map suggests that the nucleotide-binding site of the skeletal muscle myosin might be more open in the rigour state than what has been described for other actomyosin structures or rigour-like states. This could be relevant to the faster rate of ATPase cycle of skeletal muscle myosin compared with those of cytoplasmic ones.

Myosin has been called a backdoor enzyme^32^ because Pi leaves before ADP^33^ and a possible pathway for Pi release has been found only in the backside of the pocket in the myosin crystal structures^32^,^34^. However, the structure of actomyosin rigour state with such a widely open pocket (Figs 1e and 5d) suggests that Pi may also be released from the front side. Although it is not obvious why Pi leaves before ADP, electrostatic repulsion by the negative charges of Pi or the way the ADP moiety is bound by myosin may be responsible for this.

### Conformational change of myosin upon ATP binding

**Figure 6:**
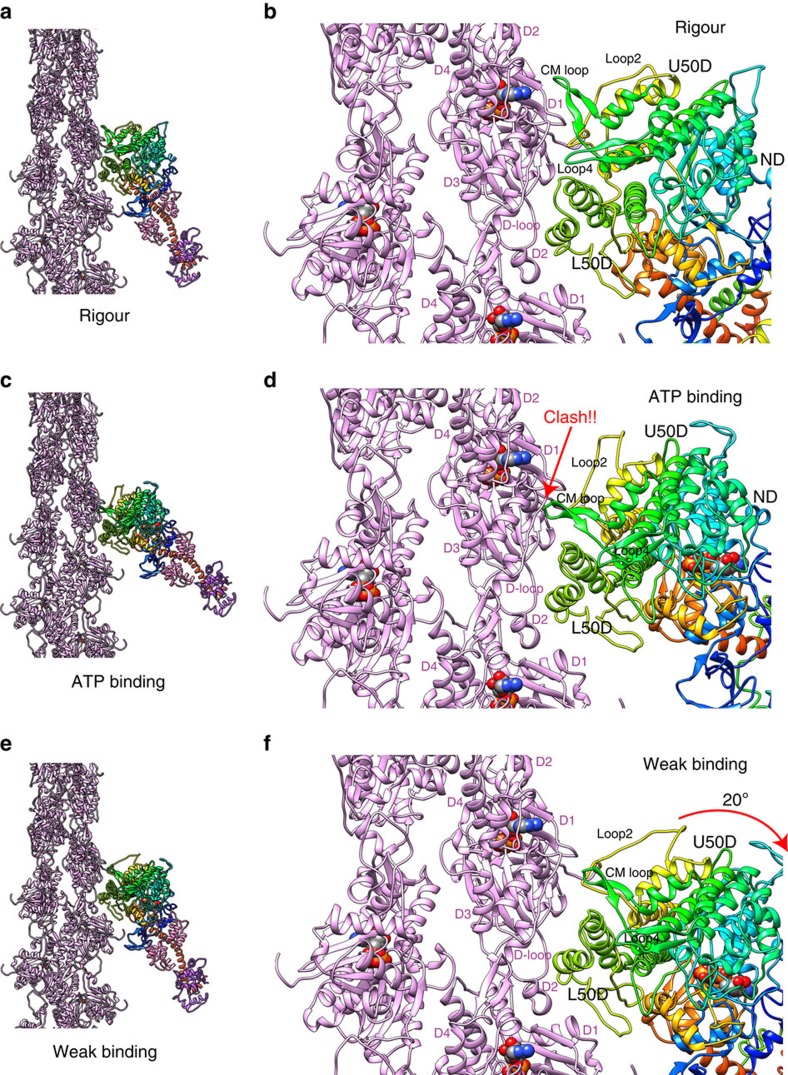
Conformational changes of rigour myosin head upon ATP binding and its possible consequence to form the weak binding state. (**a**,**b**) The actomyosin rigour structure; (**c**,**d**) myosin structure upon ATP binding with its L50D helix–loop–helix and loop 2 still attached to actin; and (**e**,**f**) after rotation of myosin head to avoid the clash of CM loop with actin where L50D helix–loop–helix and loop 2 still attached to actin. The **a**–**c** are overviews, and **b**,**d**,**f** are magnified. Note that the N-terminal portion of loop 2 must be flexible enough to allow myosin head rotation while the lysine-rich C-terminal portion stays attached to the N-terminal region of actin to keep electrostatic interactions of the weak binding state. The crystal structure of chicken muscle myosin in the post-rigour state (PDB: 2MYS)^8^ was used to build the models shown in **c**–**f** by including loop 2 in different conformations to accommodate different distances between actin D1 and myosin U50D.

We also compared our rigour model with a post-rigour structure^8^ by superposing myosin L50D (N473–A593), which contains the helix–loop–helix that is tightly attached to both actin A3 and A1 as described above (Fig. 6a–d and Supplementary Fig. 9a–d), to see what would occur in the actomyosin interactions upon ATP binding. We used L50D for superposition because this domain binds over two actin subunits and occupies the largest area of actomyosin interface. In the rigour state, CM loop and loop 4 are nicely fitted on and tightly bound to actin surface (domains D1 and D3, Figs 1d and 6b), but the post-rigour structure thus superimposed on the rigour structure shows a serious steric clash of CM loop with domain D1 of actin (Fig. 6d and Supplementary Fig. 9d) that is caused by U50D rotation nearly as a rigid body by 21° around the long axis of myosin head, tilted 40° off the actin filament axis (Supplementary Fig. 7). Hence, this clash of CM loop appears to be the main cause of myosin dissociation from actin filament upon ATP binding. Assuming that L50D and loop 2 stay bound to both actin A3 and A1 with hydrophobic and electrostatic interactions, respectively, this CM loop clash against actin would push CM loop back and cause ∼20° clockwise rotation of the entire motor domain around its long axis to avoid the clash, resulting in a marked reduction in the interacting surface area between myosin head and two actin subunits to destabilize the actomyosin interactions (Fig. 6c–f, Supplementary Fig. 9c–f and Supplementary Video 4). This model would represent a possible structure of actomyosin in the weak binding state formed upon ATP binding, and this would be the state of myosin ready to dissociate from actin filament.

### Possible structure of weak binding state before strong binding

The weak binding state of actomyosin in the ADP–Pi state is one of the distinct, biochemically well-characterized states^7^. This is the state of actomyosin ready to transform into strong binding. It is, however, difficult to experimentally visualize the structure of the weak binding state because it is not stable and the lifetime is short, only on the order of millisecond^35^. It is well established that weak binding is dominated by electrostatic interactions^36^. We can therefore envisage that the actomyosin interactions in the weak binding state formed upon ATP binding described above may also represent the weak binding state of actomyosin in the ADP–Pi state (Fig. 6e,f and Supplementary Fig. 9e,f), except that the lever arm domain should be in the primed or pre-power stroke orientation. The electrostatic interactions between the C-terminal part of myosin loop 2 and the N-terminal regions of actin, together with the flexible nature of the N-terminal portion of loop 2, would work as a flexible tether to keep myosin attached while allowing its rotation without immediate dissociation from actin filament. The hydrophobic interactions of the helix–loop–helix of myosin L50D with two consecutive actin subunits and the flexible D-loop of actin would allow this rotation as a hinge (Fig. 6f and Supplementary Movie 5). Although this weak binding structure is modelled with ATP-bound myosin in the post-rigour state, ADP–Pi-bound myosin in the pre-power stroke state should have the same actomyosin interactions because the conformations of myosin U50D and L50D in the pre-power stroke state are similar to those in the post-rigour state. This also suggests that weakly bound actomyosin should exist before its dissociation as well.

**Figure 7:**
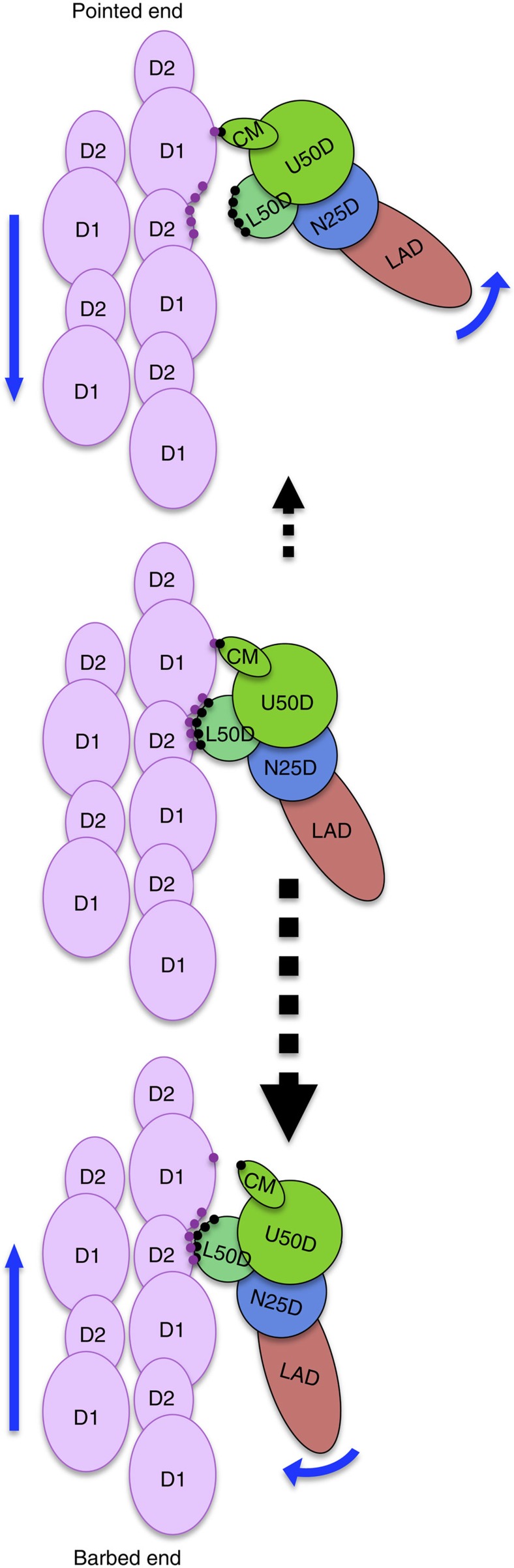
Schematic diagram of actomyosin structure in the weak binding state showing a possible mechanism of preferential transition to the strong binding state in the backward movement of actin filament (downward in this figure). Clockwise rotation of myosin by upward movement of actin filament (middle to bottom) can occur more easily than counterclockwise rotation by downward movement (middle to top), because the bonds between myosin and two actin subunits can be broken one after another by clockwise rotation, starting from those on the tip of CM loop (middle to bottom) but the tip of CM loop becomes the centre or fulcrum of rotation for further counterclockwise rotation and therefore many bonds between L50D and two actin subunits have to be broken simultaneously (middle to top). This results in a longer lifetime of the weak binding state, thereby a higher probability of transition to the strong binding state, in the backward (downward) movement of actin filament. Blue arrows indicate the directions of actin filament movement and myosin rotation, and dashed black arrows indicate the probabilities of transitions between the states by their sizes.

A preferential binding of myosin to actin filament has been observed depending on the direction of relative motion and/or force^35^. The asymmetry in the putative model of actomyosin in the weak binding state (Fig. 6e,f and Supplementary Fig. 9e,f), which is schematically depicted in Fig. 7, can explain how such a preferential binding can be achieved. As mentioned above, myosin L50D bound to two actin subunits and loop 2 bound to actin N-terminal regions can act as a hinge and a flexible tether, respectively, to allow a relatively large angle of rotation of entire myosin head around its long axis (between the middle and bottom panels of Fig. 7). When actin filament moves backward to its barbed end (downwards in Figs 6 and 7), which is opposite to the sliding direction in muscle sarcomere, the weakly bound myosin head rotates around its long axis counterclockwise as viewed from the head, resulting in the clash contact of myosin CM loop with actin (Fig. 7, middle). This contact, although nonspecific, increases the number of bonds between myosin head and actin, stabilizing their weak binding, and stops further counterclockwise rotation because this contact point becomes the rotation centre or fulcrum for further rotation that requires a large number of bonds between myosin L50D and two actin subunits to be broken almost simultaneously (Fig. 7, top). That is why backward movement of actin filament prolongs the lifetime of weak binding and increases the probability of transition from the weak to strong binding state. On the contrary, when actin filament moves forward to its pointed end (upward in Figs 6 and 7), CM loop can be easily detached from actin by clockwise rotation of myosin head (Fig. 7, bottom) because there are only a small number of nonspecific bonds in this contact, and this CM loop detachment further destabilizes the weak binding state, resulting in a much higher probability of myosin head dissociation than that in the backward movement of actin filament. Therefore, this structural asymmetry in the actomyosin interaction in their weakly bound state is likely to be key to the directional preference of transition from the weak to strong binding state (Fig. 7 and Supplementary Video 5).

We speculate that this structural asymmetry may be able to cause directionally preferential release of myosin upon ATP binding from actin filament; the probability of dissociation is higher when actin filament moves forward to its pointed end. Hence, the unidirectional sliding motions of myosin and actin filament could potentially be achieved by biasing their relative Brownian motions within each sarcomere by the preferential release of myosin toward the barbed end of actin filament. Such a thermal-driven mechanism could explain why the sliding distance of actin filament by myosin in sarcomere during one ATP hydrolysis cycle is longer than 60 nm^37^ that is much longer than the distance predicted by the power stroke of myosin lever arm. It could also explain how a single myosin head goes through multiple steps of 5.3 nm along actin filament by repeating weak binding and release from consecutive actin subunits along the long strand before strongly bound by release of ADP and Pi when myosin is forced to stay near actin filament^38^. Thermal motion-driven directionally asymmetric release would be sufficient to drive such biased motions up to some distance within a limit posed by accumulated pulling-back force, and any putative potential slope is not required. When thermal fluctuation force is balanced with pulling-back force after biased step motions over some distance, the lifetime of weak binding state becomes long enough for myosin to transform into the strong binding state.

## Methods

### Sample preparation and electron microscopy

Rabbit skeletal muscle myosin II S1 (40 μM) stored as a frozen solution at −80 °C was used to decorate F-actin. G-actin (22 μM) from rabbit skeletal muscle was polymerized in a 30 μl solution of 25 mM Hepes buffer (pH 7.5), 50 mM KCl, 1 mM MgCl_2_ and 1 mM ATP for ∼2 h at room temperature. The F-actin filaments were spun down by centrifugation at 100,000 *g* for 60 min to remove monomeric actin. The pellet was gently resuspended in polymerized buffer without ATP. Myosin and F-actin were mixed in final concentration of 13 and 6.5 μM, respectively. Apyrase was added in final concentration of 0.1 unit per ml just to completely remove ATP before the grid was made. A 2.1 μl aliquot was applied onto a grow-discharged holey carbon molybdenum grid (R0.6/1.0, Quantifoil), blotted and plunge-frozen into liquid ethane by Vitrobot (FEI). The control of temperature, 100% humidity and the timing between blotting and plunging was important to make ice-embedded myosin-decorated actin filaments as straight as possible and ice thickness as thin as possible for high-contrast, high-quality imaging. The frozen grid was observed at temperatures of 50–60 K using a JEOL JEM3200FSC electron cryomicroscope equipped with a liquid-helium cooled specimen stage, an Ω-type energy filter and a field-emission electron gun operated at 200 kV. Zero energy-loss images, with a slit setting to remove electrons of an energy loss >10 eV, were recorded on a 4 k × 4 k 15 μm per pixel slow-scan CCD camera (TemCam-F415MP, TVIPS) at an approximate magnification of × 111,000 (1.35 Å per pixel), a defocus range of 1.0–2.0 μm and an electron dose of ∼20 electrons per Å^2^. In total, 779 CCD images were collected manually in 3 days and used for image analysis.

### Image analysis

Helical image analysis was carried out using the IHRSR method^20^ with EMAN^21^ and SPIDER^22^ as previously described^23^,^24^. Defocus and astigmatism of each image were determined using a slightly modified version of CTFFIND3 (ref. 39) to prevent the effect derived from strong layer lines. Images of actin–myosin filament in 779 CCD frames were boxed into 31,535 segments of 512 × 512 pixels with a step shift of 100 pixels along the filament axis using the boxer program of EMAN^21^. The number of actin–myosin molecules used for this reconstruction corresponds to ∼120,000. Images were then corrected for a phase and amplitude contrast transfer function (CTF) by multiplying the CTF calculated from the defocus and astigmatism. We used a ratio of 7% for the amplitude CTF to the phase CTF. This procedure for the CTF correction results in the multiplication of the square of CTF (CTF^2^) to the original structure factor and suppresses the noise around the nodes of the CTF, allowing more accurate image alignment. The amplitude modification by CTF^2^ was corrected in the last stage of image analysis as described later. The images were then high-pass filtered (285 Å) to remove a density undulation of low-spatial frequency, normalized and cropped to 400 × 400 pixels. Image processing was mainly carried out with the SPIDER package^22^ on a PC cluster computer with 48 CPUs (RC server Calm2000, Real Computing, Tokyo, Japan).

A series of reference projection images were generated for each reference volume by rotating the volume azimuthally about the filament axis between 0° and 360° and projecting the volume at every 0.5° to produce all the views. The variation of the out-of-plane tilt angle was limited to ±10° and was also sampled at every 1°. The raw images of the boxed segments were translationally and rotationally aligned and cross-correlated with the set of reference projections to produce the following information: an in-plane rotation angle, an x-shift, a y-shift, an azimuthal angle and a cross-correlation coefficient for each segment. Particles with a small cross-correlation coefficient were discarded. The polarity of the particles was tracked with respect to their respective filament. Even with our high-contrast imaging technique, the orientation of each individual particle was sometime ambiguous because of the relatively low-contrast and high noise level of the segment images. Therefore, the orientation was defined as that of the majority of the segments for each filament during each alignment cycle, and all the segments identified to have the opposite orientation were discarded.

We used a solid cylinder with a diameter of 200 Å as the initial reference volume to avoid any model bias in image alignment and reconstruction. The initial helical symmetry parameters were imposed on the first reconstruction to produce the new reference volume for the second round of image alignment. After this cycle, every time a 3D image was reconstructed, the symmetry of this new volume was determined by a least-squares fitting algorithm^20^, and this symmetry was imposed upon the reconstruction. The new symmetry-enforced volume was used as a reference for the next round of alignment. This process was repeated iteratively until the symmetry values converged to a stable solution. Fourier shell correlation (FSC) was calculated at every refinement cycle, and the map was low-pass filtered at the resolution of FSC_0.5_ of the reconstruction in each refinement cycle to avoid the noise-biased overestimation. After the final refinement, segment image data sets were divided into two so that segments belonging to a filament are included in the same data set and not distributed into the two sets, and the final FSC was calculated from these two data sets. The resulting reconstruction was then modified by multiplying the transform of the reconstruction by 1/[∑CTF^2^+1/SNR] to compensate for the amplitude distortion by the contrast transfer function. The map was scaled with a B-factor of −200 Å^2^ and low-passed at 6.5 Å. The statistics of data collection and image analysis is given in Supplementary Table 1.

### Model fitting and refinement

We used the crystal structure of squid muscle myosin S1 fragment in the rigour-like state (PDB: 3I5G)^16^ and the cryoEM structure of F-actin from rabbit skeletal muscle (PDB: 3MFP)^23^ for docking and refinement. We employed DireX^25^ and FlexEM^26^ to refine these models by flexible fitting while preserving stereochemistry. We carried out this model fitting refinement carefully to avoid overfitting, by imposing a relatively strong restraint to keep the conformations of individual domains with independent hydrophobic cores unchanged as much as possible and trying not to fit individual secondary elements separately, just like we did for the actin filament structure^23^. As a reliability measure of our model, the r.m.s. deviations of Cα atoms for individual domains of myosin head of our rigour model from those of a crystal rigour-like model (PDB: 3I5G)^16^ are listed in Supplementary Table 2. The r.m.s. deviations of crystal myosin models in the post-rigour (PDB: 2MYS)^8^ and pre-power stroke (PDB: 1QVI)^30^ states as well as those of the recent cryoEM rigour model (PDB: 4A7F^19^ and 5JLH^31^) are also listed as the reference and for comparison. The r.m.s. deviations of our model from the crystal rigour-like model are all within a range from 1.0 to 1.6 Å, which are comparable to those between crystal structures, assuring that our model was refined without overfitting. The r.m.s. deviations of Cα atoms for individual domains of myosin head of our rabbit skeletal actomyosin rigour structure from those of human myosin-14 (PDB: 5JLH)^31^ are also listed in Supplementary Table 3. We made all the figures by UCSF Chimera^40^.

### Data availability

The reconstructed density was deposited to Electron Microscopy Data Bank with accession code EMD-6664 and the atomic coordinate to Protein Data Bank with PDB ID code 5H53. The data that support the findings of this study are available from the corresponding author on request.

## Additional information

**How to cite this article:** Fujii, T. & Namba, K. Structure of actomyosin rigour complex at 5.2 Å resolution and insights into the ATPase cycle mechanism. *Nat. Commun.* **8,** 13969 doi: 10.1038/ncomms13969 (2017).

**Publisher's note**: Springer Nature remains neutral with regard to jurisdictional claims in published maps and institutional affiliations.

## Supplementary Material

Supplementary Information Supplementary Figures, Supplementary Tables and Supplementary References

Supplementary Video 1 CryoEM 3D density map and fitted model of actomyosin rigor complex. This video is a magnified image of Fig. 1b, showing about eight subunits of actin and myosin head.

Supplementary Video 2 Magnified view of the cryoEM 3D density map and fitted model of actomyosin rigor complex. This is a movie version of Fig. 1c, showing two actin subunits and a myosin head.

Supplementary Video 3 Comparison of myosin head structure in the rigor state with a crystal structure of myosin in the post-rigor state (PDB: 2MYS)8. L50D (light green) is used for superposition. The nucleotide-binding pocket is widely open by counter rotations of L50D (green) and N25D (blue) when myosin head is strongly bound to actin filament in the rigor state.

Supplementary Video 4 Conformational changes of rigor myosin head upon ATP binding. The nucleotide-binding pocket is widely open in the rigor state but it closes upon ATP binding and causes the clash of myosin CM loop with actin.

Supplementary Video 5 Conformational transitions of myosin head through its association with and dissociation from actin filament coupled with ATP hydrolysis, ADP and Pi release and ATP binding.
