# Supplementary material for: Structure of actomyosin rigour complex at 5.2 Å resolution and insights into the ATPase cycle mechanism
Source: Nat Commun. 2017 Jan 9;8:13969. doi: 10.1038/ncomms13969 (PMC5227740; doi:10.1038/ncomms13969)
Supplement: Supplementary Information — Supplementary Figures, Supplementary Tables and Supplementary References [file ncomms13969-s1.pdf]

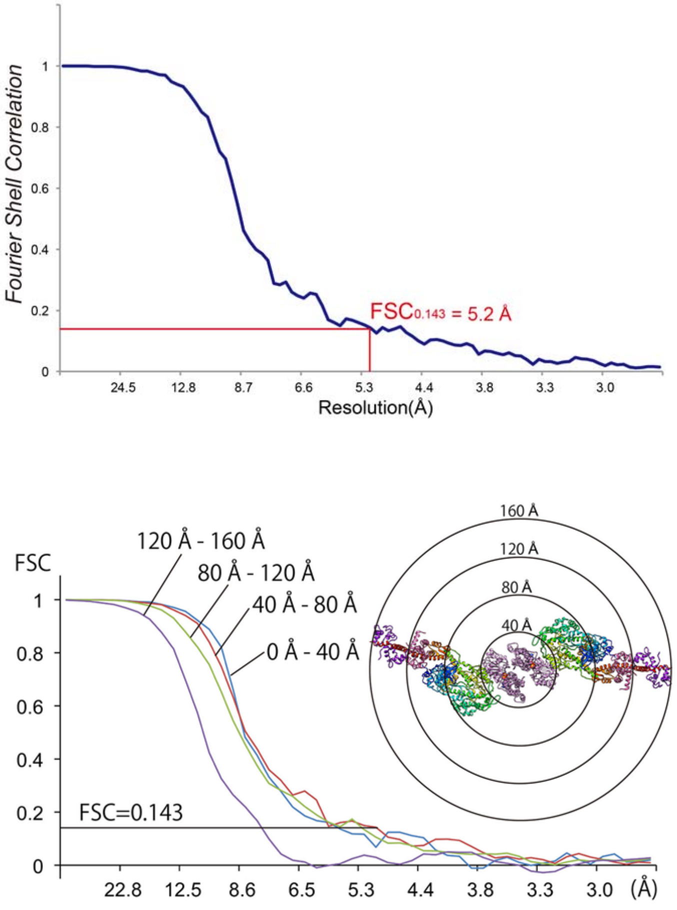

Supplementary Figure 1 Gold standard Fourier Shell Correlation (FSC) curves for the 3D reconstruction, marked with a line for an FSC of 0.143. The "gold standard" FSC was calculated from two completely independent 3D images reconstructed from the two independent sets of filament images, not from even and odd sets of image segments, to avoid the overlap in images used for analysis. The FSC of the entire structure is shown in the upper panel, and the FSCs for the four different radial regions, as indicated in the inset figure, are shown in the lower panel. The resolutions at FSC = 0.143 for the four radial regions are: 5.8 Å for 0 – 40 Å; 5.1 Å for 40 – 80 Å; 5.4 Å for 80 – 120 Å; 8.0 Å for 120 – 160 Å.

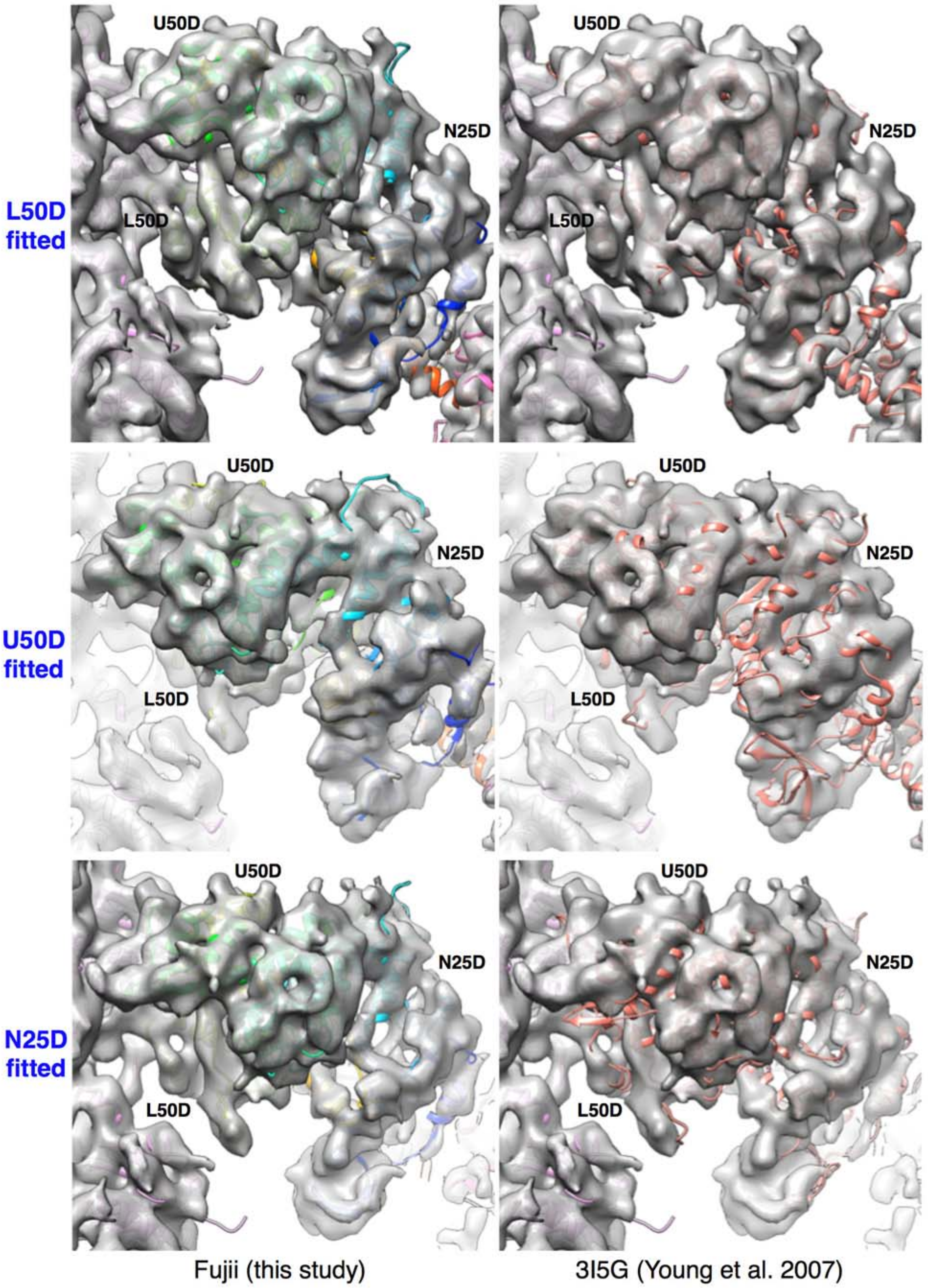

Supplementary Figure 2 Comparison of model fit to the cryoEM density map between our rigor model and a rigor-like crystal structure (PDB: 3I5G)[1]. The models are displayed in Cα ribbon representation. The rigor model is shown in rainbow in the left column, and the rigor-like crystal model in dark orange in the right column. The domains used to fit the rigor-like model are indicated on the left. The residues of each domain used for fitting are: 470 – 560 for L50D; 320 – 370 for U50D; and 150 – 200 for N25D.

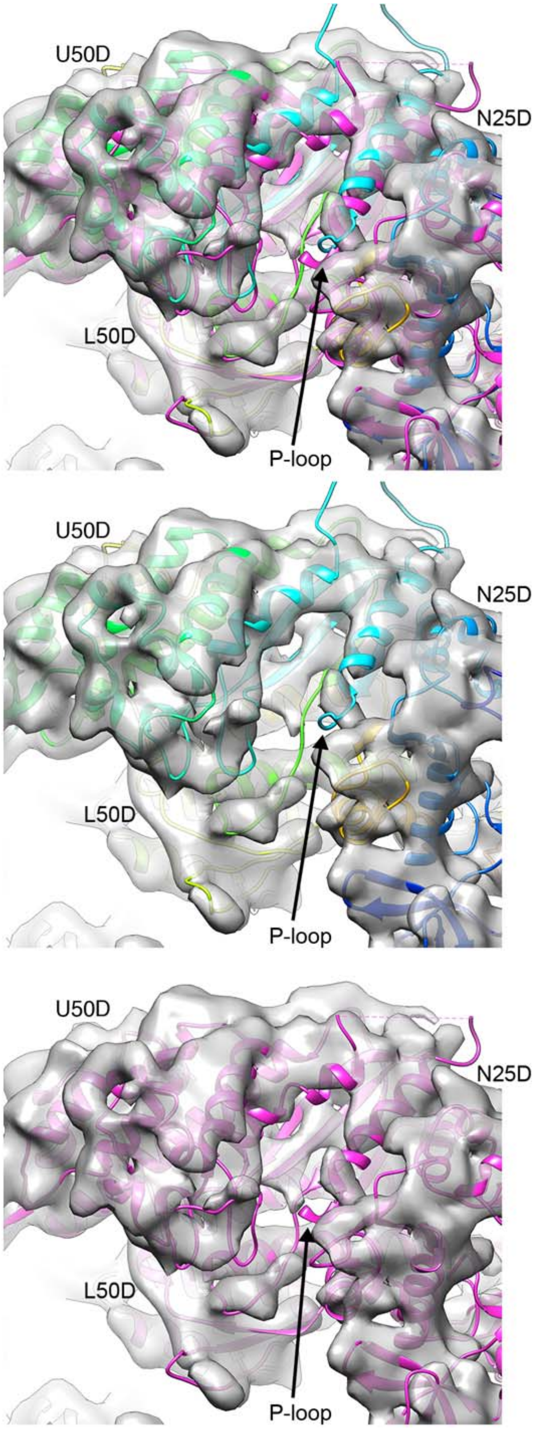

Supplementary Figure 3 Comparison of model fit to the cryoEM density map around the nucleotide-binding site between our rigor model and a rigor-like crystal structure (PDB: 3I5G)[1]. The models are displayed in $C\alpha$ ribbon representation. The rigor model is shown in rainbow and the rigor-like crystal model in magenta. Residues 470 – 560 of L50D of the rigor model are used to fit the two models.

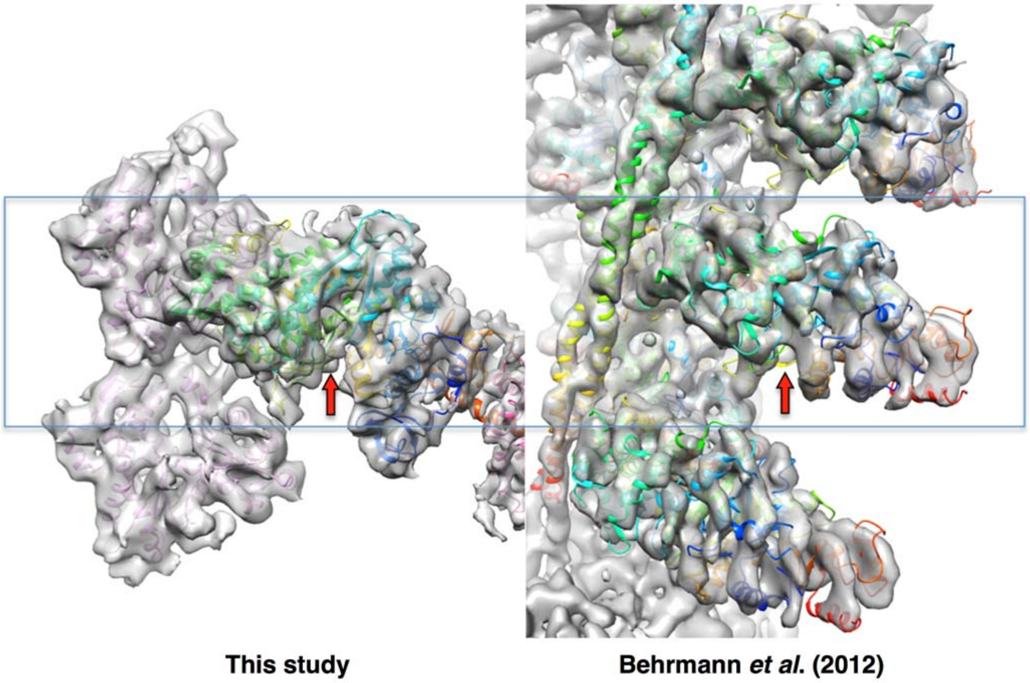

Supplementary Figure 4 Comparison of the quality of model fit to cryoEM density map between ours on the left and a previous cryoEM rigor structure (PDB: 4A7F; EMDB: EMD-1987)[2] on the right. Red arrows indicate the position of the nucleotide-binding pocket.

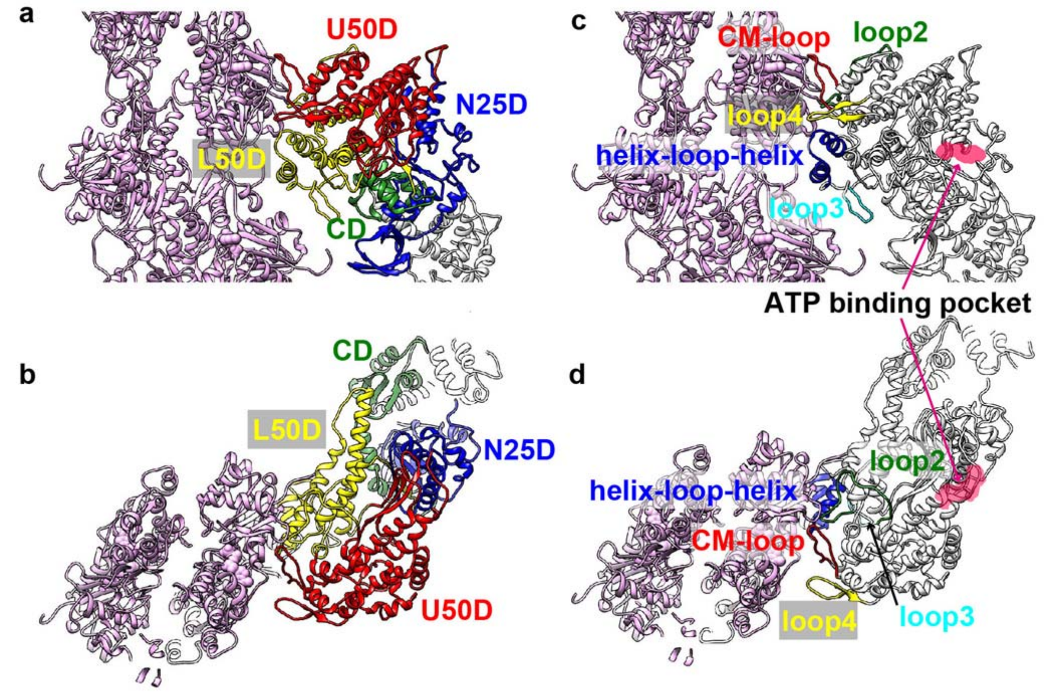

N25D: 1 – 205	loop2: 624 – 650
U50D: 206 – 466; 603 – 627	loop3: 567 – 579
L50D: 467 – 602; 628 – 680	loop4: 365 – 379
CD: 681 – 770	CM loop: 403 – 417

Supplementary Figure 5 Domains and loops of myosin colored and labeled as a guide. Actin molecules are colored purple.

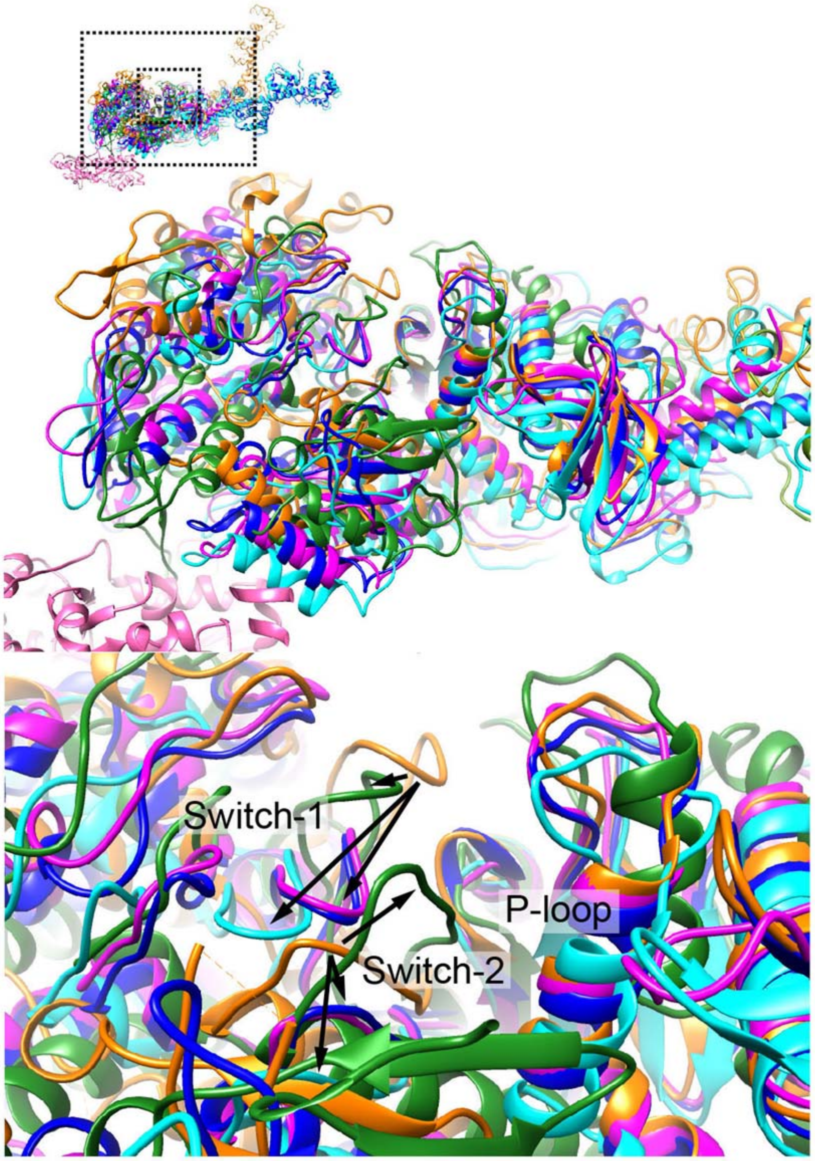

Supplementary Figure 6 Comparison of myosin structures in the actomyosin rigor state and in the rigor-like and post-rigor states in crystals. The same four models of myosin in Fig. 5 are shown together with another actomyosin rigor structure by a previous cryoEM study[2]. They are superposed with P-loop-containing strand-helix motif of N25D as in Fig. 4. The nucleotide-binding sites viewed from the barbed end of actin filament: cryoEM rigor in cyan; rigor-like (PDB: 2AKA)[3,4] in magenta; rigor-like (3I5G)[1] in blue; post-rigor (2MYS)[5] in orange; previous cryoEM rigor (4A7F)[2] in green. The small figure with dotted boxes is a guide for an enlarged overview in the middle and a further magnified view at the bottom. Actin subunits are displayed in pink to show the position and orientation of actin filament. Black arrows indicate corresponding parts of the structures between the post-rigor, rigor-like and rigor structures for Switch-1 and -2.

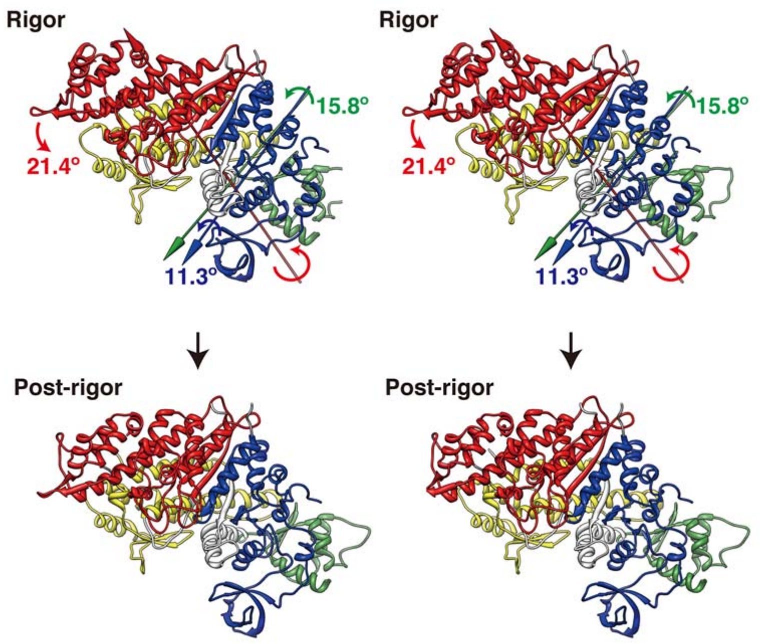

Supplementary Figure 7 Conformational changes of myosin head upon ATP binding as identified by comparison of myosin head structure in the rigor state deduced in this study (upper panel) and that of chicken muscle myosin S1 crystal structure (PDB: 2MYS)[5] regarded as post-rigor (lower panel). The models are viewed perpendicular to the axis of actin filament (side view as Fig. 1b, c and 6) in stereo.

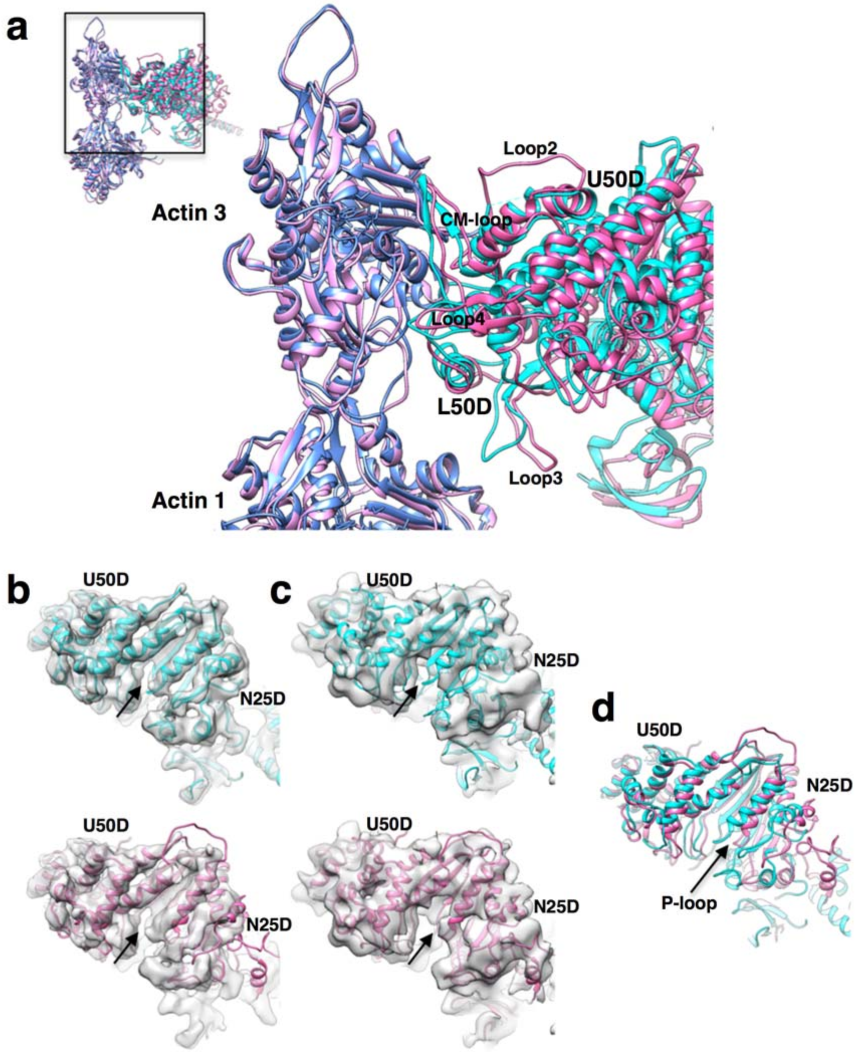

Supplementary Figure 8 Comparison of the cryoEM actomyosin rigor model of rabbit skeletal myosin 2 with that of human myosin-14 (PDB: 5JLH)[6]. Color codes are: actin in hot pink and myosin in magenta for rabbit skeletal myosin 2; and actin in blue and myosin in cyan for human myosin-14 for all the figure panels. (**a**) Actin molecules were superposed for comparison. The interactions between two actin subunits and myosin head are very similar to each other. (**b**) The two models are shown with the cryoEM actomyosin density map of human myosin-14. Domain U50D of rabbit skeletal myosin 2 (magenta) used for superposition with that of human myosin-14 shows nice fit to the density map, but its domain N25D is out of density (lower panel), indicating distinct difference in these two rigor structures. (**c**) The two models are shown with the cryoEM actomyosin density map of rabbit skeletal myosin 2. Domain U50D of human myosin-14 (cyan) shows nice fit to the density map but its domain N25D is out of density (upper panel), again indicating distinct difference in these two rigor structures. (**c**) Direct comparison of these two models by superposing U50D. The nucleotide-binding pocket indicated by short black arrows in (b) and (c) is more open in rabbit skeletal myosin 2 than human myosin-14.

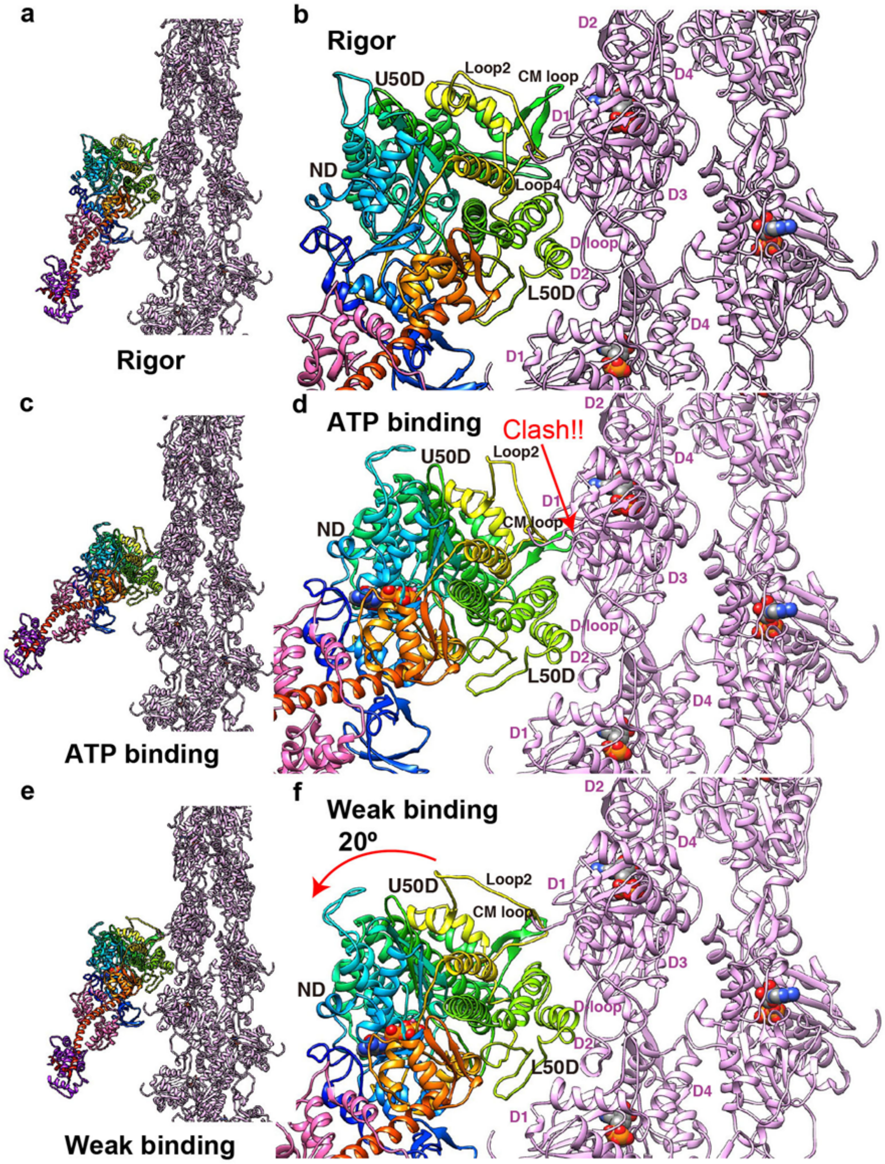

Supplementary Figure 9 Conformational changes of rigor myosin head upon ATP binding and its possible consequence to transform into the weak binding state. The structures are all the same as in Fig. 6 but viewed in the opposite direction to show the involvement of myosin loop 2 more clearly. (**a**) (**b**) The actomyosin rigor structure; (**c**) (**d**) myosin structure upon ATP binding with its L50D helix-loop-helix and loop 2 still attached to actin; and (**e**) (**f**) after rotation of myosin head to avoid the clash of CM loop with actin where L50D helix-loop-helix and loop 2 still attached to actin. **a**, **b**, **c** are overviews, and **b**, **d**, **f** are magnified.

Supplementary Table 1: Reconstruction statistics of actin-myosin rigor complex

Number of micrographs	779
Magnification	×111,294
Pixel size	1.348 Å/pixel
Total number of segmented images in initial selection	31,535
Total number of segmented images	24,055
The number of asymmetric units	117,486
Resolution (FSC = 0.143)	5.2 Å
**Helical symmetry**	
Translation	27.61Å
Rotation	166.67º

Supplementary Table 2: The root-mean-square deviations of Cα atoms for individual domains of myosin between different models

Domain (ref: 3I5G)	Rigor	2MYS	1QVI	4A7F	5JLH	
N25D-1 (12-127)	1.41	1.12	0.93	1.73	1.33	(Å)
N25D-2 (142-197)	0.99	0.66	0.79	3.58	1.06	
U50D-1 (222-352)	1.35	1.98	2.02	3.30	1.93	
U50D-2 (364-450; 606-624)	1.64	2.09	2.06	3.18	2.42	
L50D (470-490; 515-601; 646-666)	1.25	0.87	2.21	4.08	1.75	
CD (666-775)	1.53	2.86	5.66	5.76	2.20	
L50D+CD (470-490; 515-601; 646-775)	1.48	3.14	16.23	7.21	1.99	

The models compared are: crystal rigor-like model of squid myosin 2 (PDB: 3I5G)[1]; cryoEM rigor model of rabbit skeletal myosin 2 presented in this study (Rigor); post-rigor model of chicken skeletal myosin 2 (PDB: 2MYS)[5]; pre-power stroke model of scallop myosin 2 (PDB: 1QVI)[7]; cryoEM rigor model of Dictyostelium myosin 1E (PDB: 4A7F)[2]; cryoEM rigor model of human myosin-14 (PDB: 5JLH)[6]. The rms deviations were obtained by using 3I5G as a reference for superposition of individual domains of the other models. The numbers in the parenthesis under each domain indicate regions of amino acid residues. The relatively large rms deviations for CD and L50D+CD of 2MYS and 1QVI indicate the conformational differences in those domains between rigor and post-rigor and between rigor and pre-power stroke. The relatively small rms deviations for domains of cryoEM rigor models of rabbit skeletal myosin 2 (Rigor) and human myosin-14 (5JLH) from those of crystal structures indicate that the models of individual domains of these cryoEM rigor structures were properly refined by fitting them into high quality cryoEM density maps. The relatively large rms deviations of 4A7F (cryoEM rigor) from 3I5G (crystal rigor-like) suggest that the domains were largely deformed, probably by overfitting of the model to the cryoEM density map.

Supplementary Table 3: The root-mean-square deviations of Cα atoms for individual domains of myosin between the cryoEM rigor model of rabbit skeletal myosin II and human cytoplasmic myosin-14.

Domain (ref: 5JLH)	Rigor
N25D-1<br>(50-149)	2.05 (Å)
N25D-2<br>(164-219)	1.70
U50D-1<br>(236-364)	2.00
U50D-2<br>(378-464; 620-638)	2.18
L50D<br>(485-505; 530-615; 669-688)	2.23
CD<br>(689-798)	2.26
L50D+CD<br>(485-505; 530-615; 669-798)	2.35

The models compared are: cryoEM rigor models of human myosin-14 (PDB: 5JLH)[6] and rabbit skeletal myosin 2 presented in this study (Rigor). The rms deviations were obtained by using 5JLH as a reference for superposition of individual domains. The numbers in the parenthesis under each domain indicate regions of amino acid residues. The relatively small rms deviations between domains of these two structures indicate that the models of individual domains were properly refined by fitting to high quality cryoEM density maps.

Supplementary References

1 Yang, Y., Gourinath, S., Kovács, M., Mitray, L., Reutzel, R., Himmel, D. M., O'Neall-Hennessey, E., Reshetnikova, L., Szent-Györgyi, A. G., Brown, J. H. & Cohen, C. Rigor-like structures from muscle myosins reveal key mechanical elements in the transduction pathways of this allosteric motor. *Structure* 15, 553-564 (2007).

2 Behrmann, E., Müller M., Penczek, P. A., Manherz, H. G., Manstein, D. & Raunser, S. Structure of the rigor actin-tropomyosin-myosin complex. *Cell* 150, 327-338 (2012).

3 Reubold, T. F., Eschenburg, S., Becker, A., Kull, F. J. & Manstein, D. J. A structural model for actin-induced nucleotide release in myosin. *Nature Struct. Biol.* 10, 826-830 (2003).

4 Reubold, T. F., Eschenburg, S., Becker, Loonard, M, Schmid, S. L., Vallee, R. B., , Kull, F. J. & Manstein, D. J. Crystal stsructure of the GTPase domain of rat dynamin 1. *Proc. Natl. Acad. Sci. USA* 102, 1309323098 (2005).

5 Rayment, I. et al. Three-dimensional structure of myosin subfragment-1: a molecular motor. *Science* 261, 50-58 (1993).

6 von der Ecken, J., Heissler, S. M., Pathan-Chhatbar, S., Manstein, D. J. & Raunser, S. Cryo-EM structure of a hyman cytoplasmic actomyosin complex at near-atomic resolution. *Nature* **354**, 724-728 (2016).

7 Gourinath, S., Himmel, D. M., Brown, J. H., Reshetnikova, L., Szent-Györgyi, A. G., & Cohen, C. Crystal structure of scallop myosin S1 in the pre-power stroke stte to 2.6 Å resolution: flexibility and function in the head. *Structure* 11, 1621-1627 (2003).
